# Exposure of a 23F Serotype Strain of *Streptococcus pneumoniae* to Cigarette Smoke Condensate Is Associated with Selective Upregulation of Genes Encoding the Two-Component Regulatory System 11 (TCS11)

**DOI:** 10.1155/2014/976347

**Published:** 2014-06-11

**Authors:** Riana Cockeran, Jenny A. Herbert, Timothy J. Mitchell, Thérèse Dix-Peek, Caroline Dickens, Ronald Anderson, Charles Feldman

**Affiliations:** ^1^Medical Research Council Unit for Inflammation and Immunity, Department of Immunology, Faculty of Health Sciences, University of Pretoria, Private Bag X323, Pretoria 0001, South Africa; ^2^Tshwane Academic Division of the National Health Laboratory Service, 1 Modderfontein Road, Sandringham, Johannesburg 2192, South Africa; ^3^Institute of Infection, Immunity and Inflammation, College of Medical, Veterinary and Life Sciences, Glasgow Biomedical Research Centre, University of Glasgow, Sir Graeme Davies Building, 120 University Place, Glasgow G12 8TA, UK; ^4^Institute for Microbiology and Infection, School of Immunity and Infection, University of Birmingham, Birmingham B15 2TT, UK; ^5^Divisions of Haematology and Pulmonology, Department of Internal Medicine, Faculty of Health Sciences, University of the Witwatersrand and Charlotte Maxeke Johannesburg Academic Hospital, Private Bag 3,WITS, Johannesburg 2050, South Africa

## Abstract

Alterations in whole genome expression profiles following exposure of the pneumococcus (strain 172, serotype 23F) to cigarette smoke condensate (160 **μ**g/mL) for 15 and 60 min have been determined using the TIGR4 DNA microarray chip. Exposure to CSC resulted in the significant (*P* < 0.014–0.0006) upregulation of the genes encoding the two-component regulatory system 11 (TCS11), consisting of the sensor kinase, *hk11*, and its cognate response regulator, *rr11*, in the setting of increased biofilm formation. These effects of cigarette smoke on the pneumococcus may contribute to colonization of the airways by this microbial pathogen.

## 1. Introduction

The association of cigarette smoking with a predisposition to the development of microbial and viral infections of the airways is well recognized and carries a particularly high risk for invasive pneumococcal disease (IPD) [1, 2]. Although generally attributed to interference with the innate and adaptive host defences of the airways, several potentially IPD predisposing, pathogen-directed effects of cigarette smoke exposure have also been described. Notwithstanding the occurrence of a range of potential pathogens in cured tobacco [3] and the associated alterations in the composition of the microbiota of the nasopharynx [4], cigarette smoking has been reported to increase microbial virulence, predominantly by increasing the expression of adhesins and the production of biofilm [5–8]. Biofilm consists of bacterial cells encapsulated in an extracellular polymer matrix composed of DNA, proteins, and possibly polysaccharides, with choline binding proteins being intimately involved in the formation of biofilm [9]. We and others have recently reported that exposure of the pneumococcus to cigarette smoke is accompanied by increased formation of biofilm [7, 10]. However, the alterations in gene expression which precede increased formation of biofilm by smoke-exposed pneumococci have not been described. This topic is the focus of the current study.

## 2. Materials and Methods

### 2.1. Bacterial Strain

An antibiotic-sensitive clinical isolate of* Streptococcus pneumoniae* (strain 172, serotype 23F, multilocus sequence type 81), which is of importance in the South African setting, was provided by the National Institute of Communicable Diseases, Johannesburg, South Africa. Importantly, serotype 23F is one of the most common causes of IPD [11]. The strain was cultured overnight and adjusted to concentrations of either 6.14 × 10^6^ (biofilm formation) or 2 × 10^8^ (gene expression) colony-forming units (cfu)/mL in tryptone soy broth (TSB, Merck, Darmstadt, Germany) prior to exposure to cigarette smoke condensate.

### 2.2. Cigarette Smoke Condensate (CSC)

CSC (Murty Pharmaceuticals, Lexington, KY, USA) was dissolved in dimethylsulfoxide (DMSO) and used at final concentrations of 80 and 160 *μ*g/mL (biofilm production) or 160 *μ*g/mL only (gene expression). Solvent controls were included in all experiments. The total amount of condensate generated during the combustion of one cigarette is 26.3 milligrams [12].

### 2.3. Global Gene Expression

The protocols used for RNA extraction, conversion to labelled cDNA, and whole genome expression are shown as supplementary data (see supplementary data in Supplementary Material available online at http://dx.doi.org/10.1155/2014/976347). Briefly, the bacteria were exposed to either CSC dissolved in DMSO or DMSO only for either 15 or 60 min, after which they were pelleted by centrifugation and snap-frozen in liquid nitrogen. Following extraction, RNA was transcribed to cDNA and amplified by real time PCR, gene expression was detected using the* S. pneumoniae* TIGR4 DNA microarray chip (Bacterial Microarray Group (B*μ*g@S), St. George's Hospital, London, United Kingdom). Probes printed onto the microarray slide were designed based on the genome sequenced strain TIGR4, representing all 2236 open reading frames, with a further 117 probes added to represent unique genes present in the R6 genome sequence, array version SPv1.1.0 (detailed procedures described in the supplementary data). The TIGR4 microarray chip is broadly representative of the genetic profile of* S. pneumoniae* and has been used as an indicator of gene expression for other pneumococcal strains [13].

A more limited series of confirmatory relative gene expression experiments, focused specifically on those genes identified as being significantly up- or downregulated using the microarray procedure, were also performed. This procedure (included as supplementary data) is essentially similar to that described above with isolation of RNA, conversion to cDNA by RT-PCR, amplification of cDNA, and detection with real time PCR.

### 2.4. Biofilm Formation

This was performed in parallel with the gene expression studies to ensure strict comparison and was measured after 16 hours of incubation at 37°C/5% CO_2_ on bacteria adherent to the sides of 6-well tissue culture plates cultured in the presence or absence of CSC using a crystal violet (0.1%)-based spectrophotometric procedure as described previously [10].

### 2.5. Statistical Analysis

For the gene expression studies, a total of 3 biological replicas were performed per condition, using 12 microarrays (3 arrays were used for each of the control and CSC-exposed time points (15 and 60 min)). Statistical analysis of RNA expression was performed in GeneSpring using the statistical analysis (ANOVA) tool, performing a 1-way parametric test without assuming variances are equal. False discovery rate was set to 0.05 (5% gene false discovery rate), and a Benjamini and Hochberg false discovery rate multiple testing correction was applied. This resulted in the creation of lists of genes highlighting those which were significantly upregulated or down regulated in the CSC-exposed systems. The results for the CSC-treated systems are expressed as fold alteration in the levels of gene expression relative to those of the corresponding untreated control systems for each time interval.

The relative gene expression real time PCR experiments were performed using RNA from 3 different experiments, with 3 replicates in each system, comparing untreated and CSC-exposed bacteria over 15 and 60 min time intervals. Statistical analyses were performed on the quantification cycle (Cq) data, using the Wilcoxon matched pairs test, and the results were expressed as the mean ± standard error of the mean (SEM) of the log normalised relative quantities (NRQ). Data were analysed using qBase software (http://www.biogazelle.com).

In the case of biofilm formation, a total of 3 experiments with 3 to 6 replicates for each system were performed, and the results were expressed as the mean value ± SEM and the data was analysed using the Mann-Whitney *U* test.

## 3. Results

### 3.1. Microarray Analysis

**Figure 1:**
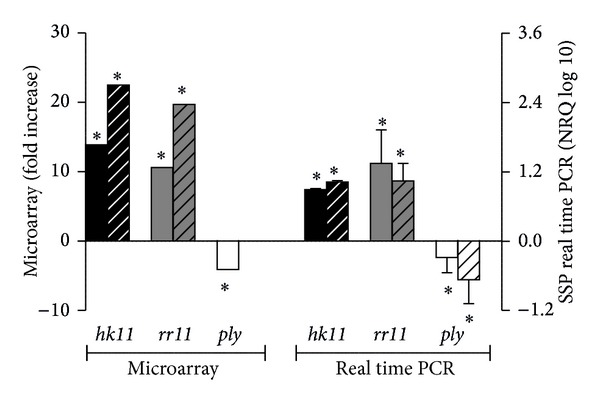
Effects of exposure of strain 172 of the pneumococcus to cigarette smoke condensate (CSC, 160 *μ*g/mL) for 15 min (solid bars) and 60 min (striped bars) on expression of the* hk11, rr11,* and* ply* genes using the* Streptococcus pneumoniae* TIGR4 microarray and selective relative gene expression procedures. The results of 3 experiments with 3 replicates in each system are expressed as either fold increase (microarrays) or normalised relative quantities (NRQ, real time PCR), respectively. *P < 0.05.

As shown in Figure 1, exposure of the pneumococcus to CSC for either 15 or 60 min resulted in selective, statistically significant upregulation of* hk11* and* rr11*. The pneumolysin gene (*ply*) was significantly downregulated after 15 min of exposure. Collectively, the* hk11* and* rr11* genes comprise the two-component regulatory system 11 (TCS11), the former being the membrane-associated histidine kinase and the latter its cognate response regulator [14]. Three other genes were upregulated at 15 min but not at 60 min: the SpTIGR4-2004 and -2005* hyp* genes (20.1+ and 17.9+, resp., *P* = 0.0137 for both) and the SpTIGR4-2003 gene (18.8+, *P* = 0.0156). No specific function has been allocated to the* hyp* (hypothetical) genes, but SpTIGR4-2003 is the ATP-binding component of an ATP-binding cassette transporter and upregulation thereof is possibly indicative of a stress response.

These findings were confirmed in an additional series of relative gene expression experiments focused exclusively on the* hk11, rr11*, and* ply* genes. These results are also shown in Figure 1.

### 3.2. Biofilm Formation

**Figure 2:**
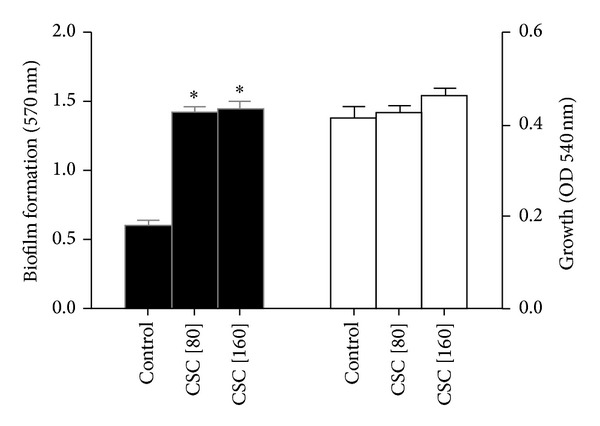
Effects of exposure of strain 172 of the pneumococcus to cigarette smoke condensate (CSC, 80 and 160 *μ*g/mL) on biofilm formation (solid black bars) and growth (solid white bars) following 16 hours of incubation. The results of 3 experiments with 3 to 6 replicates in each system are expressed as the mean values ± SEMs. *P < 0.05.

As reported previously [10], and shown in Figure 2, exposure to CSC was accompanied by a statistically significant increase in biofilm formation by the pneumococcus, independent of its effects on growth.

## 4. Discussion

The highly selective upregulation of TCS11 and its associated genes, probably part of a single operon with* rr11* belonging to the NAR subfamily of regulators [14], was found to precede the increase in biofilm formation which accompanies exposure of strain 172 of the pneumococcus to CSC. Although its function in the pneumococcus is unknown, it is noteworthy that TCS11 was first described in* Streptococcus mutans*, representing a two-component signal transduction system encoding the* hk11* and* rr11* genes, which was found to be involved in biofilm formation and acid resistance [15]. In an earlier study, deletion of the putative TCS11 homologue, 479* hk/rr*, of* S. pneumoniae* strain 0100993 (serotype 3) did not affect the numbers of viable bacteria in the lungs of mice 48 hours after intranasal infection, consistent with a limited role in bacterial virulence [14]. However, the experimental design of that study [14] is unlikely to mimic the interaction between cigarette smoke exposure, upregulation of TCS11, increased biofilm formation, and possible colonization of the airways described in the current study. Moreover, the homologue of response regulator 11 in* Bacillus cereus*, YvfTU, appears to regulate the expression of the transcriptional activator* plcR*, which is in turn a major regulator of virulence [16].

The transient downregulation of expression of the* ply* gene also observed in the current study, although interesting, is more difficult to explain. It may simply represent a redirection of cellular biosynthetic activity geared to biofilm formation. Alternatively, albeit speculatively, pneumolysin may negatively regulate biofilm formation. Several other genes were also upregulated, but the exact functions of these have not been established.

Exposure of* Staphylococcus aureus* to cigarette smoke has also been reported to result in increased biofilm formation and gene expression [8]. Genes encoding the quorum-sensing (*agr*) system which promotes biofilm dispersal were downregulated, while those encoding* sarA* and* rbf*, which promote biofilm formation, were upregulated [8]. These effects of cigarette smoke exposure were associated with transcriptional induction of antioxidative oxidoreductases and were attenuated by an antioxidant, compatible with oxidative stress as being the primary cause of smoke-mediated biofilm formation.

While the findings of the current study implicate TCS11 in biofilm formation following exposure of strain 172, serotype 23F of the pneumococcus to CSC, we do concede that the genetic basis of biofilm formation may be both strain- and stressor-dependent. In keeping with this contention, others have reported on the involvement of pneumolysin, as well as the LuxS/autoinducer 2 and Com quorum-sensing systems in spontaneous biofilm formation by strain D39 (virulent serotype 2) of the pneumococcus grown in conditions simulating the interactions of the microorganism with human respiratory epithelium [17, 18]. However, others using the virulent serotypes 4, 6A, and 6B in a murine model of colonisation of the nasal septa reported that efficient biofilm formation was dependent on the involvement of multiple factors, especially CiaRH, pneumococcal serine-rich repeat protein (PsrP), and pyruvate oxidase (SpxB), with a lesser requirement for pneumolysin, as found in the current study, and LuxS [19]. CiaRH also belongs to the 13-member family of two-component signal transduction systems of the pneumococcus and is also known as TCS05 [20].

Although the findings of the current study demonstrate upregulation of genes encoding the TCS11 of the pneumococcus, several limitations of this preliminary study preclude the establishment of a definitive relationship between this event and biofilm formation. This, in turn, is dependent on the generation of gene knockout mutants selectively targeting* hk11* and* rr11* in the setting of attenuation of CSC-mediated augmentation of biofilm formation.

In conclusion, induction of biofilm formation, possibly as stress response resulting in transcriptional activation of TCS11, may contribute to cigarette smoke-mediated colonization of the respiratory tract by the pneumococcus.

## Supplementary Material

Supplementary data: Protocols used for RNA extraction, conversion to labelled cDNA and whole genome expression.
