# Supplementary material for: Exposure of a 23F Serotype Strain of Streptococcus pneumoniae to Cigarette Smoke Condensate Is Associated with Selective Upregulation of Genes Encoding the Two-Component Regulatory System 11 (TCS11)
Source: Biomed Res Int. 2014 Jun 11;2014:976347. doi: 10.1155/2014/976347 (PMC4071851; doi:10.1155/2014/976347)
Supplement: Supplementary file 1 — Supplementary data: Protocols used for RNA extraction, conversion to labelled cDNA and whole genome expression. [file 976347.f1.docx]

**Supplementary data:** Exposure of a 23F serotype strain of *Streptococcus pneumoniae* to cigarette smoke condensate is associated with selective upregulation of genes encoding the two-component regulatory system 11 (TCS11).

### Bacterial culture and exposure

Strain 172 of the pneumococcus was cultured overnight in tryptone soy broth (Merck, Dramstadt, Germany) to a mid-log phase. The cultures were standardized using optical density to a concentration of 2 x 10^8^ cfu/ml prior to exposure to Cigarette smoke condensate (CSC) or DMSO (control system) (160 µg/ml) for 15 or 60 minutes at 37°C, 5% CO_2_. The bacterial cells were concentrated by centrifugation, the pellets snap frozen in liquid nitrogen and stored at -80°C until extraction. Three separate experiments were performed on different days, with two to three replicates for each system in each experiment.

**RNA extraction**

RNA extraction was performed in a two-step process, firstly by breaking up the bacterial cells using lysozyme (Sigma-Aldrich), glass beads (Sigma-Aldrich) and a tissue lyser (Hybaid Ribolyser [Hybaid, Cambridge, UK] for the microarrays, or TissueLyser [Qiagen, Hilden, Germany] for real time PCR); and then by isolating the bacterial RNA using the RNeasy mini kit (Qiagen) according to the manufacturer’s protocol. Any contaminating DNA was removed by treatment of the isolated RNA with either the TURBO DNA-free kit (Ambion, Austin, TX) for the microarrays or with RNase-free DNaseI (NEB, Ipswich, MA) followed by an RNA cleanup using the RNeasy mini kit (Qiagen) for real time PCR.

#### Microarray analysis

#### cDNA synthesis

cDNA was synthesized using the Superscript II Reverse Transcriptase kit (Invitrogen, Carlsbad, CA). The isolated RNA was mixed with random primers (Invitrogen) and incubated at 70°C for 10 minutes (Techgene thermal cycler, Bibby Scientific, Stone, Staffordshire, UK) before snap cooling on ice. To each sample 5μl 5x first strand buffer*, 2.5μl DTT* (100mM), 2.3μl dNTP mix (5mM dGTP/ dATP/ dTTP and 2mM dCTP, Invitrogen), 1.7μl Cy3-dCTP/ Cy5-dCTP (GE Healthcare, Chalfont St Giles, Buckinghamshire, UK) and 2.5μl Superscript II (200U/μl) were added. Samples were incubated at 25°C for 10 minutes followed by 45°C for 90 minutes.

* Supplied with with Superscript II (Invitrogen).

Post incubation a Cy3 labelled sample (control) was combined with a Cy5 labeled sample (treated system) and this cDNA sample purified using the MinElute PCR purification kit (Qiagen) as per manufacturer’s guide. Each sample was mixed with 20x SSC buffer (Ambion) and 2% SDS (20% sodium dodecyl sulphate, Ambion). Samples were incubated at 95°C for 2 minutes, briefly allowed to cool and then pipetted under the raised lifter slip (Erie Scientific Company, Portsmouth, NH) covering the microarray. Microarray slides were sealed in hybridization cassettes, submerged in water pre-warmed to 65°C and incubated overnight in the dark for 16-20 hours in a Techne Hybridiser HB-1D (Techne, Minneapolis, MN).

After incubation microarray slides were washed in 65°C pre-warmed wash A (20ml 20xSSC, 1ml 20% SDS made to 400ml with sterile distilled water) for 2 minutes with agitation, to allow the lifter slips to slide off the array slide. Slides were subsequently washed in wash B (1.2ml 20xSSC made to 400ml with sterile distilled water) for 4 minutes with agitation. Slides were centrifuged to remove any residual liquid at 1500g for 5 minutes, and stored in a dust free box until scanning.

#### Microarray analysis

Microarray technology was used to look at gene expression differences in the exposed and un-exposed pneumococcus. Microarrays (Bacterial Microarray group (Bμg@S), St. Georges Hospital, London, UK) were based on the genome of strain TIGR4, and represented all 2236 open reading frames, with a further 117 probes that were added to represent unique genes seen in the R6 genome sequence, array version SPv1.1.0. Arrays were performed in triplicate with RNA on each slide being from a different biological replicate, of each system and each time point.

Microarray slides were scanned using ScanArray Express™ (Packard biosciences, Biochip Technologies, Perkin Elmer) and the resulting TIFF (tagged image file format) images used for analysis. TIFF images were imported into Bluefuse for microarrays 3.5© (BlueGnome LTD, UK) with control data (Cy3 labelled) in channel 1 and test strain data (Cy5 labelled) in channel 2. The files containing the microarray gridmap were created and provided by BμG@S, this (SPv1_1_0­_CGH_Gridmap.bcf) automatically removes control spots from the analysis in Bluefuse. Further preliminary post processing analysis involved initial exclusion of unreliable data due to poor hybridization with a confidence estimate of below 0.1. To account for spatial, intensity and dye related effects a “Global Lowess excluding all with text”. normalization step was performed with confidence flags set at default. Replicas of each dye swap were combined by fusion.

Further data analysis was performed in Genespring GX 7.3.1 (Agilent Technologies, USA). Files created in Bluefuse (Output_fused.xls) for each of the three biological replicas were imported into Genespring 7.3.1. Initial normalization consisted of a “dye swap” and a “per gene” step. Statistical analysis of RNA expression was performed in Genespring using the statistical analysis (ANOVA) tool, performing a 1-way parametric test without assuming variances are equal. False discovery rate was set to 0.05 (5% gene false discovery rate), and a Benjamini and Hochberg false discovery rate multiple testing correction applied. This analysis was used to create lists of genes that were statistically differentially regulated between treated and un-treated systems.

**Relative quantification using real time PCR**

Validation of some of the gene expression changes seen in the microarray experiments were performed using real time PCR. RNA was extracted (see above) from the samples treated at the two different time points as well as from the untreated samples. The concentrations of the RNA extracts were determined by spectrophotometry with the NanoDrop ND-1000 Spectrophotometer and the quality of the extracts was assessed by resolving a 1 µl aliquot on an agarose gel.

### cDNA synthesis

cDNA was synthesised using the High-Capacity cDNA Reverse Transcription kit (Applied Biosystems, Foster City, CA) in accordance with the manufacturer’s instructions. Non–reverse transcribed (NRT) negative controls, prepared in an identical manner except that nuclease-free water was added to each reaction instead of reverse transcriptase, were also included.

### Real time PCR

Relative quantitative real-time PCR was performed, in duplicate for each sample, using the Stratagene Brilliant II SYBR^®^ Green QPCR low ROX master mix (Agilent, Santa Clara, CA) and CFX-96 well plates (BioRad, Hercules, CA) on a CFX96 real-time PCR detection system (Bio-Rad Laboratories Inc.,Hercules, Calif.). Each 25 ul reaction mixture contained 1 x Brilliant II SYBR® Green QPCR Low ROX Master Mix, 200 nM of each primer and 10 ng of cDNA. Thermocycling conditions for all primer pairs comprised an initial denaturation step of 95°C for 10 minutes; 40 amplification cycles of 95°C for 30 seconds and 60°C for 1 minute; and a melt curve step of 95°C for 30 seconds and 60°C for 60 seconds ramping in increments of 0.5°C. The genes assayed were Pneumolysin, *ply* (SP 1923) (5'-AGGCAGTCGCTTTACAGCAGATCA-3' and 5'-TGGGCAACATAGGCACCACTATGA-3'), the DNA-binding response regulator *rr11* (SP 2000) (5'-GCTTCAACCGGATGTAGAG-3' and 5'-TTCTGCTCGTATCCACTCC-3') and the sensor histidine kinase *hk11* (SP 2001) (5'-TTCCCCATCCTGTCTGTAG-3' and 5'-AGGCAGTATTCCCAACTACAT-3'). Gene expression was normalized against two reference genes: *DNA gyrase subunit A* (SP 1219) (5'-AATCTTGCTCATACGTGCCTCGGT-3' and 5'-ATGGTGGAGCTACCGTTACATGCT-3') and *DNA gyrase subunit B* (SP 0806) (5'-TCAGCCAAATCTGGTCGTAACCGT-3' and 5'-AATTCTGCGCCAAATCCTGTTCCC-3'). Primers for Pneumolysin and DNA gyrase subunits A and B were designed using Primer Quest (http://eu.idtdna.com/Scitools/Applications/Primerquest). Comparison of gene expression between the treated and untreated samples was performed using the 2^–ΔΔCq^ method and the qbase software (Biogazelle, Zwijnaarde, Belgium). In brief, change in quantification cycle (ΔCq) values for each sample was calculated for both the genes of interest and the reference genes by subtracting the Cq value of the treated sample from the Cq value of the untreated sample. The relative quantities (2ΔCq) of the genes of interest and the reference genes were calculated. To normalize to multiple reference genes, the geometric mean of the relative quantities of the reference genes was calculated [Hellemans *et al.* Genome Biol. 2007;8:R19]. The normalized relative quantity of each gene of interest was determined by dividing the relative quantity of the gene of interest by this geometric mean. These normalized relative quantities were log-transformed to allow for statistical analysis.
